# Probable REM Sleep Behavior Disorder Is a Risk Factor for Symptom Progression in Parkinson Disease

**DOI:** 10.3389/fneur.2021.651157

**Published:** 2021-04-07

**Authors:** Ashley Barasa, Jijia Wang, Richard B. Dewey

**Affiliations:** ^1^Department of Neurology, UT Southwestern Medical Center, Dallas, TX, United States; ^2^Department of Applied Clinical Research, Southwestern School of Health Professions, UT Southwestern Medical Center, Dallas, TX, United States

**Keywords:** Parkinson disease, REM sleep behavior disorder, progression, severity, prediction

## Abstract

**Background:** The literature is conflicting on whether rapid eye movement sleep behavior disorder (RBD) is associated with more rapid progression of Parkinson disease (PD).

**Objective:** We aimed to determine (1) how stable probable RBD (pRBD) is over time and (2) whether it predicts faster PD progression.

**Methods:** We evaluated participants in the Parkinson's Disease Biomarker Project (PDBP) who were prospectively assessed every 6–12 months with a series of motor, non-motor, disability, and health status scales. For aim 1, we calculated the incidence and disappearance rates of pRBD and compared stability of pRBD in PD with control subjects. For aim 2, we developed multiple regression models to determine if pRBD at baseline influenced the rate of change or average value at 48 months of 10 outcome variables.

**Results:** We found that pRBD was a less stable diagnosis for PD than controls. In pRBD+ subjects, the Movement Disorder Society-Sponsored Revision of the Unified Parkinson's Disease Rating Scale (MDS-UPDRS) part III score progressed 2.78 points per year faster (*p* < 0.01), MDS-UPDRS total score progressed 3.98 points per year faster (*p* < 0.01), a global composite outcome (GCO) worsened by 0.09 points per year faster (*p* = 0.02), and Parkinson's Disease Questionnaire (PDQ-39) mobility score progressed 2.57 percentage points per year faster (*p* < 0.01). The average scores at 48 months were 8.89 (*p* = 0.02) and 14.3 (*p* = 0.01) points higher for pRBD+ in MDS-UPDRS part III and total scores, respectively.

**Conclusions:** Our study confirms that pRBD detected at the start of a study portends more rapid progression of PD. Knowing this could be useful for enriching clinical trials with fast progressors to accelerate discovery of a disease modifying agent.

## Introduction

The degeneration of brain dopamine pathways is the pathophysiologic hallmark of idiopathic Parkinson disease (PD). Loss of brain dopamine is the primary basis of the cardinal motor signs which are substantially reversed by dopaminergic drug therapy. While dopamine deficiency may play a role in certain non-motor features such as depression and anxiety (1), it is now clear that PD is a multisystem degenerative disease. Rapid eye movement (REM) sleep behavior disorder (RBD) is one such non-dopaminergic feature seen commonly in PD which is characterized by dream enactment behavior. Affected patients kick, thrash, punch, and vocalize during REM sleep and may injure themselves or their bed partners (2). The pathophysiology of RBD in humans is not completely understood, but it is suspected that an inhibitory projection from the pontine sublaterodorsal nucleus to the spinal cord degenerates, thus removing the normal paralysis of skeletal muscle during REM sleep (3). The prevalence of RBD in the general population is about 1% (4) as compared with that in PD of 42.3% (5). While several studies have emphasized that RBD is a prodromal feature of the alpha synucleinopathies (6–8), in another study, 55% of PD patients with RBD developed it either at the same time PD was diagnosed or after the diagnosis was made (9).

Because the presence of RBD in PD is associated with more widespread alpha synuclein deposition (10), a number of studies have addressed the question of whether RBD is associated with a more malignant form of PD. A recent review suggested that RBD portends a poor prognosis, yet the findings of several longitudinal studies were conflicting (11). While two studies were concordant in demonstrating an increased risk of dementia when RBD was present (RBD+) (12, 13), two studies suggested that RBD is a risk factor for hallucinations (13, 14), while another found no association (15). With respect to progression of motor features, one study found an increased risk in RBD+ as compared to RBD– subjects (16), another found no difference in motor progression between the groups (15). A significant limitation of these studies was the inclusion of relatively small numbers of subjects followed for relatively short periods of time.

Recently, this same question was addressed using the longitudinal Parkinson's Progression Marker Initiative (PPMI) database of 421 drug-naïve patients with PD and 196 controls who were followed for 5 years. The authors defined motor progression as an increase of one point on the Hoehn and Yahr scale (H&Y) measured in the clinical “off” state and cognitive progression as a self-report of cognitive impairment with a Montreal Cognitive Assessment score (MoCA) of <26. They found using multivariate Cox hazard survival analysis that RBD+ was a predictor of motor progression with a hazard ratio (HR) of 1.49 and of cognitive progression with a HR of 2.0 (17).

Because a clear consensus has not yet emerged on the relationship between RBD and disease progression, we undertook evaluating this question using the Parkinson's Disease Biomarker Project (PDBP) database which consists of 416 PD subjects and 156 controls with longitudinal assessments (18, 19). All but 31 PD subjects were treated with dopaminergic drugs at study entry. The duration of follow-up was variable based on when they were enrolled during the 5 year project. The aims of this study were (1) to determine if a diagnosis of probable RBD (pRBD+) is stable during longitudinal follow-up and (2) to identify whether pRBD+ at baseline is a risk factor for motor, non-motor, or cognitive progression.

## Methods

### Standard Protocol Approvals, Registrations, and Patient Consents

The study protocol was reviewed and approved by the Institutional Review Board of the University of Texas Southwestern Medical Center and by the other institutions that collected data from subjects. Written informed consent was obtained from all participants. The study was registered on clinicaltrials.gov with registration number NCT01767818. The study is reported in accordance with STROBE reporting criteria for cohort studies.

### Subjects

PD subjects had a diagnosis of idiopathic PD according to UK Brain Bank Criteria (20), were male or female age 30 years old or older at the time of diagnosis, if untreated with dopaminergic agents had confirmation of dopamine transporter deficit by I-123 Ioflupane SPECT (DatScan), and if treated with dopaminergic agents had clinical evidence of a favorable response to treatment. Subjects were excluded if they had confirmed or suspected atypical parkinsonian syndromes due to drugs, metabolic disorders, encephalitis, or degenerative diseases. Control subjects were drawn from a convenience sample of roughly aged-matched persons without degenerative neurologic diseases in the same geographical area as the PD subjects. Many of the controls were spouses or partners of PD subjects.

### RBD Diagnosis

The American Academy of Sleep Medicine has established criteria for RBD that require the presence of REM sleep without atonia on polysomnography (PSG) (21). Because repetitive PSG is impractical for a large longitudinally followed cohort study, survey instruments have been developed to identify pRBD. We used the Mayo Sleep Questionnaire (MSQ) to identify RBD when a “yes” was given to question 1 and a “no” to question 5. Question 1 identifies those with dream enactment behavior, and question 5 excludes those with symptoms suggestive of sleep apnea. The use of these two questions is associated with a sensitivity and specificity for detecting RBD of 98% and greater than 74%, respectively (22). In this report we refer to our subjects as having pRBD due to the lack of PSG confirmation. However, it should be noted that the Diagnostic and Statistical Manual of Mental Disorders (DSM-5) defines RBD without the need for PSG in patients who have the appropriate symptoms in the context of a synucleinopathy diagnosis (23). The MSQ was obtained annually from baseline until the last visit.

The incidence rate for onset of pRBD was calculated as the number of new pRBD+ cases divided by the time elapsed in years divided by the total number of pRBD– cases at baseline. The rate of disappearance of pRBD was calculated as the number of pRBD+ cases at baseline who converted to pRBD– at the end of the study divided by the time elapsed in years divided by the number of pRBD+ cases at baseline as previously described (15).

### Clinical Assessments

Clinical assessments performed every 6 months included the Movement Disorder Society-Sponsored Revision of the Unified Parkinson's Disease Rating Scale (MDS-UPDRS) (24) and the levodopa equivalent daily dose (LEDD) calculated from detailed drug administration records available at each visit. The LEDD was calculated according to Tomlinson and others (25) as modified by incorporating the daily dose of carbidopa and levodopa extended-release capsules (Rytary) × 0.7 and carbidopa/levodopa enteral suspension (Duopa) × 0.97. We also recorded the number of subjects from each group taking on a scheduled basis at baseline dopamine agonists (pramipexole, ropinirole, and rotigotine), sedating antidepressants (trazodone and doxepin), quetiapine, benzodiazepines (clonazepam, diazepam, alprazolam, lorazepam, and elprazolam), prescription sleep aids (zolpidem and eszopiclone), and melatonin. Every 12 months the following scales were obtained: Hamilton Anxiety Scale (HAM-A) (26), Hamilton Depression Scale (HAM-D) (27), Montreal Cognitive Assessment (MoCA) (28), Parkinson's Disease Questionnaire (PDQ-39) (29), Epworth Sleepiness Scale (ESS) (30), Modified Schwab and England Activities of Daily Living Scale (S&E) (31), and University of Pennsylvania Smell Identification Test (UPSIT) (32). We also calculated a global composite outcome (GCO) which combines parts I–III of the MDS-UPDRS, S&E, and MoCA according to the method of Fereshtehnejad and others (33). Each of the above scales, including subparts of each scale, were treated as outcome variables to determine if pRBD status at baseline was associated with symptom progression. Because a change of raters at one PDBP longitudinal site introduced an anomaly in the MDS-UPDRS part III data at visits following the 12 month assessment, we censored MDS-UPDRS part III and total scores from that site at visits from 18 months onward.

### Statistical Analysis

The two-sample *t*-test and chi-square test were used for the comparison of clinical features at baseline. For aim 1, we compared the stability of pRBD over time using the Wilcoxon–Mann–Whitney test, where the stability index was calculated by dividing the total number of visits by the count of switch events +1. A switch event represents a subject switching diagnosis (pRBD– to pRBD+ and vice versa). A high stability index indicates a more stable diagnosis over time.

For aim 2, we used univariate linear regression to estimate the subject-specific rate of change per year in each outcome measure and from this predicted measurements at 48 months. We then conducted multiple regression models to identify if having pRBD at baseline affected the rate of change in the outcome variables while controlling for demographic (age, gender, education, and ethnicity) and clinical (LEDD at baseline and PD duration) variables.

All statistical analyses were carried out using SAS 9.4 (SAS Institute Inc, Cary, NC). Graphs were generated using Prism version 8 (GraphPad Software, LLC).

## Results

Three hundred thirty-seven PD subjects and 137 controls had baseline and at least one follow-up measure on the MSQ and were thus included in this analysis. As shown in Table 1, PD subjects who were pRBD+ at baseline were older, had a longer disease duration, and were taking higher doses of levodopa equivalents. Additionally, they were more likely to have motor fluctuations, olfactory dysfunction, anxiety, daytime sleepiness, and to endorse subjective difficulty with cognitive function and bodily discomfort. Regarding medication use potentially relevant to RBD, there was a higher percentage of patients taking dopamine agonists in the pRBD+ group and a lower percentage taking melatonin. No significant difference was seen in the percentage taking sedating antidepressants, quetiapine, benzodiazepines, or prescription sleep aids comparing the two groups.

**Table 1:** Baseline characteristics of controls and PD subjects by pRBD status (Mean ± Interquartile Range).

**Variable**	**Control**	**pRBD–**	**pRBD+**	***P*-value for RBD– vs. RBD+**	***P*-value for three groups**
*n*	137	229	108		
Age^*^	65.16 ± 15.0	63.97 ± 13.0	66.18 ± 9.0	0.037	0.124
Education years	15.70 ± 5.0	15.57 ± 5.0	15.79 ± 5.0	0.477	0.758
Sex (M)	45.99%	60.09%	57.41%	0.641	0.028
Hispanic or Latino	1.60%	4.21%	4.95%	0.765	0.337
Years with PD^*^		4.24 ± 5.0	6.44 ± 7.0	<0.0001	
MDS-UPDRS Part I	3.61 ± 4.0	7.95 ± 7.0	9.27 ± 6.5	0.057	<0.0001
MDS-UPDRS Part II	0.47 ± 0.0	8.86 ± 9.0	10.15 ± 11.0	0.159	<0.0001
MDS-UPDRS Part III	3.80 ± 4.0	21.14 ± 14.0	22.06 ± 15.5	0.581	<0.0001
MDS-UPDRS Part IV^*^	0.01 ± 0.0	1.94 ± 3.0	2.99 ± 6.0	0.009	<0.0001
MDS-UPDRS Total	7.90 ± 7.0	39.90 ± 28.0	44.47 ± 30.5	0.128	<0.0001
MoCA	26.23 ± 4.0	25.79 ± 4.0	25.69 ± 5.0	0.832	0.379
LEDD^*^		572.97 ± 466.0	704.73 ± 515.0	0.012	
Schwab and England	0.99 ± 0.0	0.88 ± 0.1	0.87 ± 0.1	0.513	<0.0001
PDQ-39 Mobility	1.17 ± 0.0	13.23 ± 17.5	15.44 ± 17.5	0.334	<0.0001
PDQ-39 ADL	0.94 ± 0.0	16.04 ± 20.8	17.45 ± 20.8	0.488	<0.0001
PDQ-39 Emotional	4.93 ± 8.3	13.20 ± 20.8	14.25 ± 16.6	0.545	<0.0001
PDQ-39 Stigma	0.36 ± 0.0	14.59 ± 25.0	12.85 ± 18.8	0.41	<0.0001
PDQ-39 Social Support	2.43 ± 0.0	5.84 ± 8.3	6.46 ± 8.3	0.688	0.011
PDQ-39 Cog. Impair^*^	5.11 ± 6.2	15.80 ± 18.8	22.19 ± 25.0	0.001	<0.0001
PDQ-39 Communication	1.89 ± 0.0	12.92 ± 16.7	15.11 ± 25.0	0.301	<0.0001
PDQ-39 bodily discomfort^*^	11.92 ± 16.7	21.44 ± 33.3	26.87 ± 25.0	0.027	<0.0001
GCO	−0.60 ± 0.2	0.19 ± 0.7	0.32 ± 0.7	0.164	<0.0001
HAM-A^*^	3.88 ± 4.0	6.17 ± 6.0	7.55 ± 6.0	0.03	<0.0001
HAM-D	2.66 ± 4.0	4.78 ± 5.0	5.24 ± 5.0	0.376	<0.0001
Epworth sleepiness^*^	5.09 ± 4.0	7.24 ± 6.0	8.32 ± 6.0	0.047	<0.0001
UPSIT^*^	32.60 ± 6.0	20.38 ± 13.0	18.28 ± 8.0	0.02	<0.0001
Dopamine agonists^*^		51.53%	53.70%	<0.0001	
Sedating antidepressants		3.06%	4.63%	0.564	
Quetiapine		2.62%	4.63%	0.763	
Benzodiazepines		6.55%	14.81%	0.858	
Melatonin^*^		3.06%	0%	0.016	
Sleep aids		2.18%	0.93%	0.219	

* *Indicates statistically significant pRBD+ vs. pRBD– (p < 0.05)*.

*PD, Parkinson disease; MoCA, Montreal Cognitive Assessment; PDQ-39, Parkinson's Disease Questionnaire; GCO, Global Composite Outcome; HAM-A, Hamilton Anxiety Scale; HAM-D, Hamilton Depression Scale; UPSIT, University of Pennsylvania Smell Identification Test*.

Of the pRBD– PD subjects at baseline, the incidence rate of conversion to pRBD+ was 8.7% as compared to that in controls of 1.2% (*p* < 0.001). By contrast, the disappearance rate in PD subjects was 12.7% as compared to 17.5% in controls (ns). The mean pRBD stability index was 2.62 for PD and 3.25 for controls (*p* < 0.001) indicating that pRBD is a significantly less stable diagnosis in PD subjects.

**Figure 1:**
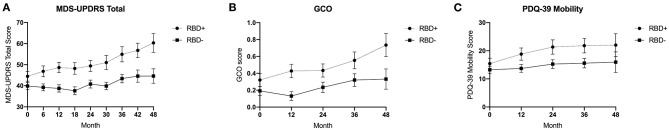
Multivariate linear regression of **(A)** MDS-UPDRS total score, **(B)** global composite outcome, and **(C)** PDQ-39 mobility subscale over 48 months comparing pRBD+ and pRBD– PD subjects. Error bars represent standard error of the mean.

Table 2 and Figure 1 show the results of the multivariate linear regression analysis evaluating whether pRBD+ status at baseline is associated with symptom progression as measured by a series of outcome measures. These models were adjusted by age, sex, years of education, ethnicity, LEDD at baseline, and years with PD. The results were that MDS-UPDRS part III and total scores, GCO, and PDQ-39 mobility score progressed more rapidly in pRBD+ PD subjects as compared to those who were pRBD– at baseline.

**Table 2:** Multiple linear regression analysis showing the difference in progression rate (defined as rate of change per year) as measured by various clinical endpoints (estimate represents pRBD+ at baseline minus pRBD– subjects).

**Outcome**	**Estimate**	**95% CI for estimate**	***P*-value**	**R-squared for goodness of fit**
MDS-UPDRS Part I	0.46798	(−0.13, 1.06)	0.1227	0.035
MDS-UPDRS Part II	0.32196	(−0.40, 1.05)	0.3827	0.032
MDS-UPDRS Part III^*^	2.77659	(0.78, 4.77)	0.0065	0.038
MDS-UPDRS Part IV	−0.1759	(−0.59, 0.24)	0.4025	0.043
MDS-UPDRS Part total^*^	3.98442	(1.26, 6.71)	0.0043	0.055
MoCA	0.17412	(−0.14, 0.49)	0.2756	0.042
LEDD	−10.721	(−44.05, 22.60)	0.5272	0.110
Schwab and England	−0.01	(−0.02, 0.00)	0.1564	0.017
GCO^*^	0.08874	(0.01, 0.17)	0.0234	0.051
Epworth Sleepiness Scale	0.14562	(−0.28, 0.57)	0.5019	0.033
UPSIT	0.37048	(−0.26, 1.00)	0.248	0.045
HAM-A	−0.3563	(−1.00, 0.29)	0.2784	0.043
HAM-D	−0.156	(−0.60, 0.29)	0.492	0.074
PDQ-39 Mobility^*^	2.57433	(0.69, 4.46)	0.0076	0.039
PDQ-39 ADL	1.12134	(−0.49, 2.74)	0.1727	0.041
PDQ-39 Emotional	0.77624	(−1.07, 2.62)	0.4092	0.023
PDQ-39 Stigma	0.49178	(−1.60, 2.58)	0.6438	0.010
PDQ-39 Social Support	0.6179	(−0.98, 2.22)	0.4471	0.050
PDQ-39 Cognitive Impairment	1.17589	(−0.77, 3.13)	0.2363	0.020
PDQ-39 Communication	1.68476	(−0.21, 3.58)	0.0815	0.020
PDQ-39 bodily discomfort	2.29803	(−0.01, 4.60)	0.0508	0.053

* *Indicates statistically significant (p < 0.05)*.

*PD, Parkinson disease; MoCA, Montreal Cognitive Assessment; PDQ-39, Parkinson's Disease Questionnaire; GCO, Global Composite Outcome; HAM-A, Hamilton Anxiety Scale; HAM-D, Hamilton Depression Scale; UPSIT, University of Pennsylvania Smell Identification Test*.

The difference between pRBD+ and pRBD– average scores at 48 months computed from subject-specific univariate linear regression are shown in Table 3. pRBD+ at baseline was associated with significantly higher average MDS-UPDRS part III and total scores and PDQ-39 mobility, cognitive impairment, and bodily discomfort scores at 48 months.

**Table 3:** Multiple linear regression analysis showing the difference in average measurement at month 48 for each outcome measure (estimate represents pRBD+ at baseline minus pRBD– subjects).

**Outcome**	**Estimate**	**95% CI for estimate**	***P*-value**	**R-squared for goodness of fit**
MDS-UPDRS Part I	2.09154	(−0.29, 4.48)	0.0854	0.040
MDS-UPDRS Part II	1.39865	(−1.67, 4.47)	0.3704	0.151
MDS-UPDRS Part III^*^	8.89219	(1.14, 16.64)	0.0247	0.069
MDS-UPDRS Part IV	−0.3562	(−1.82, 1.11)	0.6327	0.063
MDS-UPDRS Part total^*^	14.2595	(3.14, 25.37)	0.0121	0.130
MoCA	1.13738	(−0.20, 2.47)	0.0942	0.140
LEDD	−7.2608	(−136.87, 122.35)	0.9123	0.233
Schwab and England	−0.0303	(−0.09, 0.03)	0.2852	0.081
GCO	0.28528	(−0.03, 0.60)	0.0775	0.167
Epworth Sleepiness Scale	1.07744	(−0.59, 2.75)	0.2051	0.073
UPSIT	0.60998	(−1.95, 3.17)	0.6393	0.114
HAM-A	−0.8171	(−3.27, 1.64)	0.5131	0.019
HAM-D	−0.6758	(−2.39, 1.04)	0.4386	0.075
PDQ-39 Mobility^*^	8.8832	(0.86, 16.91)	0.0302	0.144
PDQ-39 ADL	2.73898	(−3.96, 9.44)	0.4216	0.152
PDQ-39 Emotional	3.26148	(−3.37, 9.90)	0.3342	0.034
PDQ-39 Stigma	−0.4285	(−7.89, 7.04)	0.9101	0.021
PDQ-39 Social Support	2.16916	(−3.37, 7.71)	0.4416	0.035
PDQ-39 Cog. Impairment^*^	8.42905	(1.33, 15.53)	0.0201	0.043
PDQ-39 Communication	4.57143	(−2.70, 11.84)	0.217	0.074
PDQ-39 Bodily Discomfort^*^	10.2371	(2.01, 18.47)	0.0149	0.118

* *Indicates statistically significant (p < 0.05)*.

*PD, Parkinson disease; MoCA, Montreal Cognitive Assessment; PDQ-39, Parkinson's Disease Questionnaire; GCO, Global Composite Outcome; HAM-A, Hamilton Anxiety Scale; HAM-D, Hamilton Depression Scale; UPSIT, University of Pennsylvania Smell Identification Test*.

## Discussion

Our analysis of the PDBP dataset of prospectively assessed PD subjects produced two major findings. First, we confirmed previous reports that pRBD is not a stable condition in PD when assessed longitudinally by survey instruments (15, 16, 34). In our subjects, about 9–13% reported onset or resolution of pRBD during follow up. Interestingly, a recent 3 year study of PD patients with known RBD found that while subjective ratings of RBD symptoms increased, decreased, or remained stable, REM sleep without atonia as shown by PSG increased over time in all subjects (35). This suggests that patient or bed partner ratings of dream enactment behavior are not particularly reliable indicators of RBD when measured repeatedly and that once the pathology underlying RBD has developed, it progresses over time. We speculate that the fluctuating responses on the MSQ in subjects with pRBD are related to treatment effects, bed partner attentiveness to the problem, amnesia for these events by patients, and random variability of symptoms.

Second, we found that pRBD at baseline was a risk factor both for rate of worsening of several outcomes and of worse average scores at 48 months. This was true for both objective motor scores and several subscales of the PDQ-39 health status measure. Taken together, these data support the developing consensus that RBD is a marker of more extensive underlying neurodegeneration (36, 37).

Limitations of our study include missing values for MDS-UPDRS part III at one of three sites caused by a change in the clinical rater. This reduced the amount of available data for this outcome measure at later time points and highlights the importance of maintaining a consistent rater for motor scales in longitudinal investigations, even when, as in this case, all raters were certified in performing the MDS-UPDRS following the training program provided by the International Parkinson and Movement Disorder Society. Another limitation was the use of the MSQ for identification of RBD, which as noted earlier, is less reliable than PSG. Our use of this questionnaire potentially underestimated the number of subjects diagnosed with RBD because only those with clinically significant symptoms were identified. Fewer controls were recruited as compared to PD subjects because the priority for the PDBP project was to collect clinical and biospecimens on individuals with PD. Finally, the follow-up time varied among subjects with those entering the study in the first year of recruitment having longer follow-up than those recruited later during the project. This problem was managed by using univariate linear regression to model the subject-specific rate of progression of each outcome variable through 48 months. The strengths of our study are the longitudinal design, prospective data collection, the large number of subjects, and the availability of age-matched controls.

Our results add to the growing body of literature indicating that pRBD is linked to more rapid worsening of both motor and health status metrics when present in PD. This finding is important because clinically significant pRBD can be detected using simple patient/sleep partner survey instruments and the pRBD+ status suggests that a patient is likely to be a fast progressor. This knowledge, in turn, will be useful not only for more accurate individual prognostication in the clinic but may help investigators select subjects who are destined to be fast progressors for inclusion in disease modifying research trials.

## Data Availability Statement

Publicly available datasets were analyzed in this study. This data can be found at: https://pdbp.ninds.nih.gov/how-to-guide#request-access-to-the-dmr.

## Ethics Statement

This study involving human participants was reviewed and approved by the Institutional Review Board of the University of Texas Southwestern Medical Center, and by the other institutions that collected data from subjects. The participants provided their written informed consent to participate in this study.

## Author Contributions

AB: research project—organization and execution, statistical analysis—review and critique, and manuscript—writing of the first draft. JW: research project—execution, statistical analysis—design and execution, manuscript—review and critique. RD: research project—conception, organization, and execution, statistical analysis—review and critique, manuscript—review and critique. All authors contributed to the article and approved the submitted version.

## Conflict of Interest

RD reports personal fees (consulting) from Amneal, Acorda, Supernus, Teva, Adamas, US WorldMeds, Acadia, and Lundbeck, outside the submitted work. The remaining authors declare that the research was conducted in the absence of any commercial or financial relationships that could be construed as a potential conflict of interest.

## References

[1] Maricle RA Nutt JG Valentine RJ Carter JH. Dose-response relationship of levodopa with mood and anxiety in fluctuating Parkinson's disease: a double-blind, placebo-controlled study. Neurology. (1995) 45:1757–60. 10.1212/WNL.45.9.1757 7675241

[2] Schenck CH Bundlie SR Ettinger MG Mahowald MW. Chronic behavioral disorders of human REM sleep: a new category of parasomnia. Sleep. (1986) 9:293–308. 10.1093/sleep/9.2.293 3505730

[3] Boeve BF Silber MH Saper CB Ferman TJ Dickson DW Parisi JE. Pathophysiology of REM sleep behaviour disorder and relevance to neurodegenerative disease. Brain. (2007) 130:2770–88. 10.1093/brain/awm056 17412731

[4] Haba-Rubio J Frauscher B Marques-Vidal P Toriel J Tobback N Andries D. Prevalence and determinants of rapid eye movement sleep behavior disorder in the general population. Sleep. (2018) 41:zsx197. 10.1093/sleep/zsx197 29216391

[5] Zhang X Sun X Wang J Tang L Xie A. Prevalence of rapid eye movement sleep behavior disorder (RBD) in Parkinson's disease: a meta and meta-regression analysis. Neurol Sci. (2017) 38:163–70. 10.1007/s10072-016-2744-1 27770275

[6] Galbiati A Verga L Giora E Zucconi M Ferini-Strambi L. The risk of neurodegeneration in REM sleep behavior disorder: a systematic review and meta-analysis of longitudinal studies. Sleep Med Rev. (2019) 43:37–46. 10.1016/j.smrv.2018.09.008 30503716

[7] Postuma RB Iranzo A Hu M Högl B Boeve BF Manni R. Risk and predictors of dementia and parkinsonism in idiopathic REM sleep behaviour disorder: a multicentre study. Brain. (2019) 142:744–59. 10.1093/brain/awz030 30789229 PMC6391615

[8] Schenck CH Boeve BF Mahowald MW. Delayed emergence of a parkinsonian disorder or dementia in 81% of older men initially diagnosed with idiopathic rapid eye movement sleep behavior disorder: a 16-year update on a previously reported series. Sleep Med. (2013) 14:744–8. 10.1016/j.sleep.2012.10.009 23347909

[9] De Cock VC Vidailhet M Leu S Texeira A Apartis E Elbaz A. Restoration of normal motor control in Parkinson's disease during REM sleep. Brain. (2007) 130:450–6. 10.1093/brain/awl363 17235126

[10] Postuma RB Adler CH Dugger BN Hentz JG Shill HA Driver-Dunckley E. REM sleep behavior disorder and neuropathology in Parkinson's disease. Mov Disord. (2015) 30:1413–7. 10.1002/mds.26347 26265105

[11] Kim Y Kim YE Park EO Shin CW Kim HJ Jeon B. REM sleep behavior disorder portends poor prognosis in Parkinson's disease: a systematic review. J Clin Neurosci. (2018) 47:6–13. 10.1016/j.jocn.2017.09.019 29102236

[12] Nomura T Inoue Y Kagimura T Nakashima K. Clinical significance of REM sleep behavior disorder in Parkinson's disease. Sleep Med. (2013) 14:131–5. 10.1016/j.sleep.2012.10.011 23218532

[13] Postuma RB Bertrand JA Montplaisir J Desjardins C Vendette M Romenets SR. Rapid eye movement sleep behavior disorder and risk of dementia in Parkinson's disease: a prospective study. Mov Disord. (2012) 27:720–6. 10.1002/mds.24939 22322798

[14] Sinforiani E Pacchetti C Zangaglia R Pasotti C Manni R Nappi G. REM behavior disorder, hallucinations and cognitive impairment in Parkinson's disease: a two-year follow up. Mov Disord. (2008) 23:1441–5. 10.1002/mds.22126 18512749

[15] Lavault S Leu-Semenescu S Tezenas du Montcel S Cochen de Cock V Vidailhet M Arnulf I. Does clinical rapid eye movement behavior disorder predict worse outcomes in Parkinson's disease? J Neurol. (2010) 257:1154–9. 10.1007/s00415-010-5482-y 20148335

[16] Bugalho P Viana-Baptista M. REM sleep behavior disorder and motor dysfunction in Parkinson's disease–a longitudinal study. Parkinsonism Relat Disord. (2013) 19:1084–7. 10.1016/j.parkreldis.2013.07.017 23928300

[17] Pagano G De Micco R Yousaf T Wilson H Chandra A Politis M. REM behavior disorder predicts motor progression and cognitive decline in Parkinson disease. Neurology. (2018) 91:e894–905. 10.1212/WNL.0000000000006134 30089615

[18] Gwinn K David KK Swanson-Fischer C Albin R St. Hillaire-Clarke C Sieber BA. Parkinson's disease biomarkers: perspective from the NINDS Parkinson's Disease Biomarkers Program. Biomark Med. (2017) 11:451–73. 10.2217/bmm-2016-0370 28644039 PMC5619098

[19] Rosenthal LS Drake D Alcalay RN Babcock D DuBois Bowman F Chen-Plotkin A. The NINDS Parkinson's disease biomarkers program. Mov Disord. (2016) 31:915–23. 10.1002/mds.26438 26442452 PMC4824671

[20] Hughes AJ Daniel SE Kilford L Lees AJ. Accuracy of clinical diagnosis of idiopathic Parkinson's disease: a clinico-pathological study of 100 cases. J Neurol Neurosurg Psychiatry. (1992) 55:181–4. 10.1136/jnnp.55.3.181 1564476 PMC1014720

[21] American Academy of Sleep Medicine. International Classification of Sleep Disorders: Diagnostic and Coding Manual (ICSD-2). 2nd ed. Westchester, IL: American Academy of Sleep Medicine (2005).

[22] Boeve BF Molano JR Ferman TJ Smith GE Lin S-C Bieniek K. Validation of the Mayo Sleep Questionnaire to screen for REM sleep behavior disorder in an aging and dementia cohort. Sleep Med. (2011) 12:445–3. 10.1016/j.sleep.2010.12.009 21349763 PMC3083495

[23] American Psychiatric Association. Diagnostic and Statistical Manual of Mental Disorders (DSM-5). 5th ed. Arlington, VA: American Psychiatric Association (2013).

[24] Goetz CG Tilley BC Shaftman SR Stebbins GT Fahn S Martinez-Martin P. Movement Disorder Society-sponsored revision of the Unified Parkinson's Disease Rating Scale (MDS-UPDRS): scale presentation and clinimetric testing results. Mov Disord. (2008) 23:2129–70. 10.1002/mds.22340 19025984

[25] Tomlinson CL Stowe R Patel S Rick C Gray R Clarke CE. Systematic review of levodopa dose equivalency reporting in Parkinson's disease. Mov Disord. (2010) 25:2649–53. 10.1002/mds.23429 21069833

[26] Hamilton M. The assessment of anxiety states by rating. Br J Med Psychol. (1959) 32:50–5. 10.1111/j.2044-8341.1959.tb00467.x 13638508

[27] Hamilton M. A rating scale for depression. J Neurol Neurosurg Psychiatry. (1960) 23:56–62. 10.1136/jnnp.23.1.56 14399272 PMC495331

[28] Nasreddine ZS Phillips NA Bedirian V Charbonneau S Whitehead V Collin I. The Montreal Cognitive Assessment, MoCA: a brief screening tool for mild cognitive impairment. J Am Geriatr Soc. (2005) 53:695–9. 10.1111/j.1532-5415.2005.53221.x 15817019

[29] Peto V Jenkinson C Fitzpatrick R. PDQ-39: a review of the development, validation and application of a Parkinson's disease quality of life questionnaire and its associated measures. J Neurol. (1998) 245(Suppl. 1):S10–4. 10.1007/PL00007730 9617716

[30] Johns MW. Reliability and factor analysis of the Epworth Sleepiness Scale. Sleep. (1992) 15:376–81. 10.1093/sleep/15.4.376 1519015

[31] Ramaker C Marinus J Stiggelbout AM Van Hilten BJ. Systematic evaluation of rating scales for impairment and disability in Parkinson's disease. Mov Disord. (2002) 17:867–76. 10.1002/mds.10248 12360535

[32] Doty RL Shaman P Kimmelman CP Dann MS. University of Pennsylvania Smell Identification Test: a rapid quantitative olfactory function test for the clinic. Laryngoscope. (1984) 94:176–8. 10.1288/00005537-198402000-00004 6694486

[33] Fereshtehnejad SM Zeighami Y Dagher A Postuma RB. Clinical criteria for subtyping Parkinson's disease: biomarkers and longitudinal progression. Brain. (2017) 140:1959–76. 10.1093/brain/awx118 28549077

[34] Gjerstad MD Boeve B Wentzel-Larsen T Aarsland D Larsen JP. Occurrence and clinical correlates of REM sleep behaviour disorder in patients with Parkinson's disease over time. J Neurol Neurosurg Psychiatry. (2008) 79:387–91. 10.1136/jnnp.2007.116830 17557796

[35] Figorilli M Marques AR Vidal T Delaby L Meloni M Pereira B. Does REM sleep behavior disorder change in the progression of Parkinson's disease? Sleep Med. (2020) 68:190–8. 10.1016/j.sleep.2019.12.013 32044557

[36] Rahayel S Gaubert M Postuma RB Montplaisir J Carrier J Monchi O. Brain atrophy in Parkinson's disease with polysomnography-confirmed REM sleep behavior disorder. Sleep. (2019) 42:zsz062. 10.1093/sleep/zsz062 30854555 PMC6559168

[37] Salsone M Cerasa A Arabia GM Gambardella A Mumoli L. Reduced thalamic volume in Parkinson disease with REM sleep behavior disorder: volumetric study. Parkinsonism Relat Disord. (2014) 20:1004–8. 10.1016/j.parkreldis.2014.06.012 24998995

